# Supplementary material for: Protocol for the systematic review of the epidemiology of superficial Streptococcal A infections (skin and throat) in Australia
Source: PLoS One. 2021 Aug 11;16(8):e0255789. doi: 10.1371/journal.pone.0255789 (PMC8357163; doi:10.1371/journal.pone.0255789)
Supplement: S1 Appendix — (DOCX) [file pone.0255789.s001.docx]

S1 Appendix: Search Strategy

| MEDLINE | Search 1:   - Subject headings (with all subheadings included)   - Streptococcal infections/   - exp Impetigo/   - Pyoderma/   - Skin Diseases, Infectious/   - Streptococcus pyogenes/   - exp Pharyngitis/   - exp nasopharyngitis/   - exp Tonsillitis/   - Respiratory Tract Infections/   - Bacterial skin diseases/ - Text words   - pharyngit*.tw.   - (nasopharyngit* or rhinopharyngit*).tw.   - tonsillit*.tw.   - (tonsil* adj2 (inflam* or infect*)).tw.   - ((throat* or pharyn*) adj3 (infect* or inflam* or strep*)).tw.   - (sore* adj2 throat*).tw.   - Skin sore*.tw.   - Group A strep*.tw.   - Streptococ*.tw.   - Impetigo.tw.   - pyoderma.tw.   **AND**  Search 2:   - Subject headings (including all subheadings)   - exp australia/   - Australian capital territory/   - New south wales/   - Northern territory/   - queensland/   - South australia/   - tasmania/   - victoria/   - Western australia/   - Oceanic Ancestry Group/   - Vulnerable populations/ - Text words   - australia*.tw.   - Melbourne.tw.   - Sydney.tw.   - perth.tw.   - Brisbane.tw.   - Canberra.tw.   - Hobart.tw.   - Darwin.tw.   - Cairns.tw.   - Adelaide.tw.   - kimberl*.tw.   - Arnhem.tw.   - Aboriginal.tw.   - Indigenous.tw.   **AND**  Search 3:   - Subject headings   - Focus groups/   - Surveys and questionnaires/   - exp Incidence/   - exp Prevalence/   - Disease notification/   - Epidemiologic studies/   - Cross sectional studies/   - Retrospective studies/   - Prospective studies/   - Epidemiological monitoring/   - Sentinel surveillance/   - Statistics as topic/ - Keywords (title)   - Incidence OR prevalence OR risk or rate OR epidemiology OR burden OR trend OR survey OR surveillance OR cross-section* OR longitudinal OR cohort OR prospective OR retrospective   Year 1970 - 2020 |
| --- | --- |
| Web of Science | ‘Web of Science Core Collection’  Search 1:  ALL=(Impetigo OR pyoderma OR pharyngitis OR “sore throat” OR tonsil* OR “skin sore” OR “skin sores” OR “skin infection*” OR “skin disease*” OR “strep* throat” OR “upper respiratory infection” OR “Strep* pyogenes” OR “infectious skin disease*” OR “bacterial skin disease*” OR “group A strep*” OR “strep* infection*”)  **AND**  Search 2:  ALL=(austral* OR “new south wales” OR victoria OR queensland OR tasmania OR “northern territory” OR “top end” OR NSW OR VIC OR NT OR SA OR TAS OR QLD OR ACT OR melbourne OR sydney OR perth OR brisbane OR canberra OR hobart OR darwin OR cairns OR adelaide OR kimberl* OR Arnhem OR Aboriginal OR “oceanic ancestry group” OR indigenous)  **AND**  Search 3:  TI = (Incidence OR prevalence OR risk OR rate OR epidemiolog* OR burden OR trend OR prospective OR retrospective OR Survey OR surveillance OR “cross section*” OR cross-section* OR longitudinal OR cohort OR “clinical stud*” OR “clinical trial*” OR observational OR meta-analysis OR “disease notification*” OR “systematic review”)  Year 1997 - 2020 |
| Cinahl Plus | Search 1:  Subject headings:   - Streptococcal infections/ - Impetigo/ - Pyoderma/ - Pharyngitis/ - exp Tonsillitis/   Keywords/ Title/ Abstract   - TX pharyngit* - TX (nasopharyngit* or rhinopharyngit*) - TX tonsillit* - TX (tonsil* N2 (inflam* or infect*)) - TX ((throat* or pharyn*) N3 (infect* or inflam* or strep*)) - TX (sore* N2 throat*) - TX Skin N1 sore* - TX Group N1 A N1 strep* - TX Streptococ* - TX Impetigo - TX pyoderma - TX bacterial N2 skin N2 infection   **AND**  Search 2:  Subject headings:   - exp australia/ - Australian capital territory/ - New south wales/ - Northern territory/ - queensland/ - South australia/ - tasmania/ - victoria/ - Western australia/ - Aboriginal Australians/ - Indigenous health/ - Indigenous peoples/   Keyword/ abstract/ title:   - TX australia - TX Melbourne - TX Sydney - TX perth - TX Brisbane - TX Canberra - TX Hobart - TX Darwin - TX Cairns - TX Adelaide - TX kimberl* - TX Arnhem - TX Aboriginal - TX Indigenous - TX (Aboriginal or indigenous) N2 Australia*   **AND**  Search 3:  Subheadings   - exp Epidemiology/ - Biosurveillance/ - Community assessment/ - Disease surveillance/ - Population surveillance/ - Disease registries/ - Incidence/ - Prevalence/ - Prospective studies/ - Cross-sectional studies/   Keywords (title)   - TI(Incidence OR prevalence OR risk or rate OR epidemiology OR burden OR trend OR survey OR surveillance OR cross-section* OR longitudinal OR cohort OR prospective OR retrospective)   Year 1970 - 2020 |
| Embase | Search 1:  Subheadings (all subheadings included)   - Streptococcus infection/ - Group a streptococcal infection/ - Streptococcus pyogenes/ - exp Impetigo/ - exp Pyoderma/ - Skin infection/ - Bacterial skin disease/ - exp Streptococcal pharyngitis/ - Rhinopharyngitis/ - exp Pharyngitis/ - exp Tonsillitis/ - Upper respiratory tract infection/   Keywords/ Abstract/ Title   - pharyngit*.tw,kw. - (nasopharyngit* or rhinopharyngit*).tw,kw. - tonsillit*.tw,kw. - (tonsil* adj2 (inflam* or infect*)).tw,kw. - ((throat* or pharyn*) adj3 (infect* or inflam* or strep*)).tw,kw. - (sore* adj2 throat*).tw,kw. - Skin adj sore*.tw,kw. - Group adj A adj strep*.tw,kw. - Streptococ*.tw,kw. - Impetigo.tw,kw. - pyoderma.tw,kw.   **AND**  Search 2:  Subheadings:   - exp australia/ - Australian capital territory/ - New south wales/ - Northern territory/ - queensland/ - South australia/ - tasmania/ - victoria/ - Western australia/ - Oceanic Ancestry Group/ - Vulnerable population/ - Indigenous australian/ - Indigenous people/   Keywords:   - australia*.tw. - Melbourne.tw. - Sydney.tw. - perth.tw. - Brisbane.tw. - Canberra.tw. - Hobart.tw. - Darwin.tw. - Cairns.tw. - Adelaide.tw. - kimberl*.tw. - Arnhem.tw. - Aboriginal.tw. - Indigenous.tw.   **AND**  Search 3:  Subheadings   - Health survey/ - exp Epidemiology/ - exp Incidence/ - exp Epidemiological data/ - exp Prevalence/ - Disease notification/ - Sentinel surveillance/   Keywords (title)   - Incidence OR prevalence OR risk or rate OR epidemiology OR burden OR trend OR survey OR surveillance OR cross-section* OR longitudinal OR cohort OR prospective OR retrospective |
| Global Health | Search 1:  Subject headings:   - Group A streptococci.sh. - Streptococcus pyogenes/ - exp impetigo/ - exp pyoderma/ - exp pharyngitis/ - exp tonsillitis/ - Upper respiratory tract infections.sh.   Text words   - pharyngit*.tw. - (nasopharyngit* or rhinopharyngit*).tw. - tonsillit*.tw. - (tonsil* adj2 (inflam* or infect*)).tw. - ((throat* or pharyn*) adj3 (infect* or inflam* or strep*)).tw. - (sore* adj2 throat*).tw. - Skin sore*.tw. - Group A strep*.tw. - Streptococ*.tw. - Impetigo.tw. - pyoderma.tw.   **AND**  Search 2:  Subject headings:   - exp australia/ - Australian capital territory/ - New south wales/ - Northern territory/ - queensland/ - South australia/ - tasmania/ - victoria/ - Western australia/ - Indigenous people/   aborigines/  Text words:   - australia*.tw. - Melbourne.tw. - Sydney.tw. - perth.tw. - Brisbane.tw. - Canberra.tw. - Hobart.tw. - Darwin.tw. - Cairns.tw. - Adelaide.tw. - kimberl*.tw. - Arnhem.tw. - Aboriginal.tw. - Indigenous.tw. - (Aboriginal or indigenous) adj2 Australia*.tw.   **AND**  Search 3:   - Subheadings:   - exp epidemiology/   - Disease prevalence/   - Disease statistics/   - Disease surveys/   - Epidemiological surveys   - Exp incidence/   - Sentinel surveillance/   - Cohort studies/ - Keywords (title)   - Incidence OR prevalence OR risk or rate OR epidemiology OR burden OR trend OR survey OR surveillance OR cross-section* OR longitudinal OR cohort OR prospective OR retrospective   Year 1970 - 2020 |
| Cochrane | Search 1: (ti/ab/kw)  (“Impetigo” OR “pyoderma” OR “pharyngitis” OR “sore throat” OR “tonsil*” OR "skin sore" OR “skin sores” OR “skin infection*” OR “skin disease*” OR "strep throat" OR "upper respiratory infection" OR “strep* throat” OR “Strep* pyogenes” OR “infectious skin diseases” OR “group A strep*” OR “streptococcal infection*”)  **AND**  Search 2: (ti,ab,kw)  (austral* OR “new south wales” OR victoria OR queensland OR tasmania OR “northern territory” OR “top end” OR NSW OR VIC OR NT OR SA OR TAS OR QLD OR ACT OR melbourne OR sydney OR perth OR brisbane OR canberra OR hobart OR darwin OR cairns OR adelaide OR kimberley OR Arnhem OR Aboriginal OR “oceanic ancestry group” OR indigenous)  **AND**  Search 3: (title/abstract)  (Incidence OR prevalence OR risk OR rate OR epidemiology OR burden OR trend OR prospective OR retrospective OR Survey OR surveillance OR “cross section*” OR cross-section* OR longitudinal OR cohort OR “clinical stud*” OR “clinical trial*” OR observational OR meta-analysis OR “disease notification*”) |
| Scopus | Search 1:  TI TLE-ABS-KEY(Impetigo OR pyoderma OR pharyngitis OR “sore* N/2 throat*” OR tonsil* OR (tonsil* N/2 (inflam* or infect*)) OR “skin N/1 sore” OR “skin sores” OR “skin infection*” OR “skin disease*” OR “strep* throat” OR “upper respiratory infection” OR  (nasopharyngit* or rhinopharyngit*) OR “Strep* pyogenes” OR “infectious skin disease*” OR “bacterial skin disease*” OR “group A strep*” OR “strep* infection*” OR ((throat* or pharyn*) N/3 (infect* or inflam* or strep*)))  **AND**  Search 2:  TITLE-ABS-KEY(austral* OR “new south wales” OR victoria OR queensland OR tasmania OR “northern territory” OR “top end” OR NSW OR VIC OR NT OR SA OR TAS OR QLD OR ACT OR melbourne OR sydney OR perth OR brisbane OR canberra OR hobart OR darwin OR cairns OR adelaide OR kimberl* OR Arnhem OR Aboriginal OR “oceanic ancestry group” OR indigenous OR ((Aboriginal or indigenous) N/2 Australia*))  **AND**  Search 3:  TITLE(Incidence OR prevalence OR risk OR rate OR epidemiolog* OR burden OR trend OR prospective OR retrospective OR Survey OR surveillance OR “cross section*” OR cross-section* OR longitudinal OR cohort OR “clinical stud*” OR “clinical trial*” OR observational OR meta-analysis OR “disease notification*” OR “systematic review”)  Years 1970 - 2020 |
| Grey literature | Google Scholar  Search 1: (“Impetigo” OR “pyoderma” OR “pharyngitis” OR “sore throat” OR “tonsil*” OR "skin sore" OR “skin sores”) AND Australia AND (Incidence OR prevalence OR epidemiology)  First 1000 results screened, sorted by relevance  Clinicaltrials.gov  Search 1:  (Impetigo OR pyoderma OR skin sores OR pharyngitis OR streptococcal tonsillitis OR tonsillitis OR bacterial skin disease) AND Australia  WHO IRIS library database  Search 1:  (Impetigo OR pyoderma OR pharyngitis OR tonsillitis OR streptococcal) AND Australia AND (Incidence OR prevalence OR risk OR rate OR epidemiology OR burden OR trend)  Trove  Search 1:  (Impetigo OR "sore throat" OR pyoderma OR pharyngitis OR tonsillitis OR "skin sore" OR "skin sores") AND Australia AND (Incidence OR prevalence OR epidemiology) NOT cancer  1970 - 2020  English  Australian  Research Data Australia  Search 1:  (Impetigo OR pyoderma OR pharyngitis OR tonsil* OR “skin sore” OR “skin sores” OR “strep* throat” OR “Strep* pyogenes” OR “group A strep*” OR “strep* infection*”) AND (Incidence OR prevalence OR risk OR rate OR epidemiolog* OR burden OR trend)  Subject heading: Medical and health sciences  the Grey Literature Report  Search 1: Australia AND (Impetigo OR skin sore* OR pyoderma OR pharyngitis OR streptococc* OR tonsillitis) |
