# Supplementary material for: Protocol for the systematic review of the epidemiology of superficial Streptococcal A infections (skin and throat) in Australia
Source: PLoS One. 2021 Aug 11;16(8):e0255789. doi: 10.1371/journal.pone.0255789 (PMC8357163; doi:10.1371/journal.pone.0255789)
Supplement: S2 Appendix — (DOCX) [file pone.0255789.s002.docx]

S2 Appendix: Data extraction points and definitions

| General information | Title  Authors  Date of publication   - If only year given, 01/01 of that year used   Critical appraisal |
| --- | --- |
| Timing | Duration   - T = 0 is a once off screen, cross sectional. - T> 0 is a screen overtime   Start   - If only year provided, input as 01/01 of the year stated   End   - If only the year provided, input as 31/12 of the year stated |
| Location | Country  State/ territory  Region  City/ community  ASCG region  Study site/ setting |
| Study design | Type of study  Sampling method  Qualifications of person collecting data  Population type  Number of participants |
| Population demographics | Lower age of population   - If non stated documented as ‘0’.   Upper age of population   - If none stated documented as ‘75+’.   Number of males in population  Number of Aboriginal and/or Torres Strait Islander people   - If study setting was in a remote Aboriginal community, all participants were assumed to be of Aboriginal and/or Torres Strait Islander descent. |
| Environmental/ social factors | Climate   - Koppen Classification System   Definition, exposure/ non-exposure data:   - Crowding - Dwelling characteristics and facilities - Education - Employment - Income - Nutrition - Socioeconomic status - Swimming - Other |
| Skin infection | Definition of sGAS skin infection  Specification of Strep A (Y/N)  Number of participants screened  Participants with impetigo  Number of presentations  Presentations with impetigo  Lesion number   - Documented (Y/N) - Average - Number with less than 5 - Number with 5 or more - Number with 20 or more   Lesion type   - Number with flat/ dry lesion - Number with crusted/ scabbed lesion - Number with moist lesion   Lesion distribution   - Number with only upper limbs - Number with only lower limbs - Number with only upper and lower limbs - Number with other (incl. head)   Microbiology   - Number tested for microbiology - Type of sampling - Number with Streptococcal A   Other disease   - Number with concurrent scabies - Number with underlying  chronic skin disease - Number with precipitating skin lesion identified - Number with concurrent throat disease   Infection by age  Other statistics |
| Throat disease | Definition of sGAS throat infection  Number of participants screened  Participants with throat disease  Number of presentations  Presentations with throat disease  Microbiology   - Number with sample taken for microbiology - Type of sample taken - Number with Streptococcal A   Other disease   - Number with concurrent impetigo   Infection by age  Other statistics |
