# Supplementary material for: Protocol for the systematic review of the epidemiology of superficial Streptococcal A infections (skin and throat) in Australia
Source: PLoS One. 2021 Aug 11;16(8):e0255789. doi: 10.1371/journal.pone.0255789 (PMC8357163; doi:10.1371/journal.pone.0255789)
Supplement: S3 Appendix — (DOCX) [file pone.0255789.s003.docx]

S3 Appendix: Joanna Briggs Institute Critical Appraisal Checklist for Studies Reporting Prevalence Data

1. Was the sample frame appropriate to address the target population?  (Yes/ No/ Unclear/ N/A)
2. Were study participants sampled in an appropriate way? (Yes/ No/ Unclear/ N/A)
3. Was the sample size adequate? (Yes/ No/ Unclear/ N/A)
4. Were the study subjects and setting described in detail? (Yes/ No/ Unclear/ N/A)
5. Was the data analysis conducted with sufficient coverage of the identified sample? (Yes/ No/ Unclear/ N/A)
6. Were valid methods used for the identification of the condition? (Yes/ No/ Unclear/ N/A)
7. Was the condition measured in a standard, reliable way for all participants? (Yes/ No/ Unclear/ N/A)
8. Was there appropriate statistical analysis? (Yes/ No/ Unclear/ N/A)
9. Was the response rate adequate, and if not, was the low response rate managed appropriately? (Yes/ No/ Unclear/ N/A)
10. Overall Appraisal (Include/ Exclude/ Seek further info)

Additional datapoint by the project team:

1. Overall study quality (Very poor/ Poor/ Fair/ Fair to good/ Good)
