# Supplementary material for: Protocol for the systematic review of the epidemiology of superficial Streptococcal A infections (skin and throat) in Australia
Source: PLoS One. 2021 Aug 11;16(8):e0255789. doi: 10.1371/journal.pone.0255789 (PMC8357163; doi:10.1371/journal.pone.0255789)
Supplement: Attachment — Submitted filename: SR sGAS - Response to Reviewers.docx [file pone.0255789.s006.docx]

**Response to Reviewers**

Firstly, thank you for your input and feedback on this paper. The suggestions have been taken on board and can be viewed in the ‘Revised Manuscript with Track Changes’ document.

In response to the specific reviewer comments:

**Reviewer 1**

**In line 103, there seems to be a repetition of the ideas in two consecutive sentences. They could try to avoid these repetitions.**

A portion of this statement has been deleted to better summaries the point and the introduction.

**In line 134, it is stated that there is no risk of country-based location bias. Despite this, Australia is a large country, and it would be necessary to discuss whether there is a risk of bias based on the different territories of the country**

The country-based location bias statement has been updated. It now states that “Although all papers are based in Australia, there may be regional differences in publication rates based on states and territories leading to region specific location bias. The geographical distribution of selected papers will be clearly defined, and detail areas of dense and sparse literature coverage that may contribute to bias.”

**Reviewer 2**

**In assessing study quality, why not consider measures that systematically evaluate bias in the design and implementation of the studies? Such as the Cochrane’s risk of bias Tool?**

For this systematic review we have chosen to use the Joanna Briggs Institute critical appraisal checklist for risk of bias assessment. The full checklist is documented in the Appendix 3 of the protocol paper and systematically covers multiple areas of bias.

The justification of this choice can be supported by the systematic review of the quality assessment of prevalence studies by Migliavaca et al. which states “among the currently available tools specific for prevalence studies, the Joanna Briggs Institute Prevalence

Critical Appraisal Tool has a higher methodologic rigor and addresses what we consider the most important items related to the methodological quality of prevalence studies and may be considered the most appropriate tool”. We have changed the paper to include this reference in support of the choice.

**Is it standard practice to exclude studies on the basis of bias? Why not exclude the, only in sensitivity analysis?**

Sensitivity analysis will consider overall quality of study as well as definitions for disease. Initial analysis will include all articles.

**The terms time lag bias and duplication bias are unfamiliar to me. Please provide references for their use.**

Both are according to the Cochrane Handbook for Systematic Reviews of Interventions.

Time-lag bias: “The *rapid* or *delayed* publication of research findings, depending on the nature and direction of the results.”

Duplication bias: “The *multiple* or *singular* publication of research findings, depending on the nature and direction of the results.”

Isabelle Boutron, Matthew J Page, Julian PT Higgins, Douglas G Altman, Andreas Lundh, Asbjørn Hróbjartsson; on behalf of the Cochrane Bias Methods Group. Cochrane handbook for systematic reviews of interventions, Chapter 7: Considering bias and conflicts of interest among the included studies. Cochrane Training. 2021; Part 2 (7).

**Provide justification for why you’re not considering fixed effects models.**

A random effects model is deemed appropriate given studies in this field sample from diverse populations, even though we are restricting to Australian studies.

**Provide citation for the climate classification system and a brief justification for your choice.**

“The most widely used climate classification system.” - Bailey, Robert G. “Ecosystem Geography: From Ecoregions to Sites” Springer, New York 2009, p.65

Citations included:

1. Köppen, W. Versuch einer Klassifikation der Klimate, vorzugsweise nach ihren Beziehungen zur Pflanzenwelt. *Geogr. Z.* 1900, *6*, 593-611.

2. Kottek, M.; Grieser, J.; Beck, C.; Rudolf, B.; Rubel, F. World map of the Köppen-Geiger climate classification updated. *Meteorol. Z.* 2006, *15*, 256-263.

**Will study selection be done in duplicate with a system for breaking ties?**

Yes. We have added a statement at line 161 to this effect.

“Data points will be compared and discrepancies resolved between data extractors or a third party reviewer.”

**The introduction section is lengthy and can be summarized.**

The discussion on outcomes following sGAS infection in the introduction has been reduced and can be appreciated in the newly submitted paper.

**Study years – please update to end of 2020 at the latest.**

The protocol years have been updated to reflect papers up to the end of 2020.

**Other:1. In assessing study quality, why not consider measures that systematically evaluate bias in the design and implementation of the studies? Such as the Cochran’s risk of bias Tool?
2. Is it standard practice to exclude studies on the basis of bias? Why not exclude the, only in sensitivity analysis?
3. The terms time lag bias and duplication bias are unfamiliar to me. Please provide references for their use.
4. Provide justification for why you’re not considering fixed effects models.
5. Provide citation for the climate classification system and a brief justification for your choice.
6. Will study selection be done in duplicate with a system for breaking ties?
7. The introduction section is lengthy and can be summarized.
8. Study years – please update to end of 2020 at the latest.-In line 103, there seems to be a repetition of the ideas in two consecutive sentences. They could try to avoid these repetitions.
-in line 134, it is stated that there is no risk of country-based location bias. Despite this, Australia is a large country, and it would be necessary to discuss whether there is a risk of bias based on the different territories of the country.-In line 103, there seems to be a repetition of the ideas in two consecutive sentences. They could try to avoid these repetitions.
-in line 134, it is stated that there is no risk of country-based location bias. Despite this, Australia is a large country, and it would be necessary to discuss whether there is a risk of bias based on the different territories of the country.-In line 103, there seems to be a repetition of the ideas in two consecutive sentences. They could try to avoid these repetitions.
-in line 134, it is stated that there is no risk of country-based location bias. Despite this, Australia is a large country, and it would be necessary to discuss whether there is a risk of bias based on the different territories of the country.**

**Report also according to MOOSE guidelines**

The protocol for the systematic review has been changed to also meet the criteria set by the MOOSE guidelines. A copy of the MOOSE guideline with documentation of page and line numbers of where these criteria are met has also been uploaded. The systematic review, when produced, will adhere to both PRISMA and MOOSE guidelines.

**Exclusion criteria – consider excluding studies reporting less than 20 participants**

A very good point. We did account for sample size in a non-specific way within the Joanna Briggs Institute critical appraisal checklist, but I agree that a paper with a sample size less than 20 will not be relevant to this review, therefore have added this point to the exclusion criteria.
